# Proenkephalin A 119–159 predicts early and successful liberation from renal replacement therapy in critically ill patients with acute kidney injury: a post hoc analysis of the ELAIN trial

**DOI:** 10.1186/s13054-022-04217-4

**Published:** 2022-10-31

**Authors:** Thilo von Groote, Felix Albert, Melanie Meersch, Raphael Koch, Christian Porschen, Oliver Hartmann, Deborah Bergmann, Peter Pickkers, Alexander Zarbock

**Affiliations:** 1 Department of Anaesthesiology, Intensive Care and Pain Medicine, University Hospital Münster, Münster, Germany; 2 Institute of Biostatistics and Clinical Research, University of Münster, Münster, Germany; 3 SphingoTec GmbH, Hennigsdorf, Germany; 4 Department of Intensive Care Medicine, Radboud University Medical Center, Nijmegen, The Netherlands

**Keywords:** Acute kidney injury, AKI, Biomarker, ELAIN, Renal replacement therapy, Proenkephalin A, penKid

## Abstract

**Background:**

Renal replacement therapy (RRT) remains the key rescue therapy for critically ill patients with severe acute kidney injury (AKI). However, there are currently no tools available to predict successful liberation from RRT. Biomarkers may allow for risk stratification and individualization of treatment strategies. Proenkephalin A 119–159 (penKid) has been suggested as a promising marker of kidney function in the context of AKI, but has not yet been evaluated for RRT liberation in critically ill patients with AKI.

**Methods:**

This post hoc analysis included 210 patients from the randomized clinical ELAIN trial and penKid levels were measured in the blood of these patients. Competing risk time-to-event analyses were performed for pre-RRT penKid at initiation of RRT and in a landmark analysis at day 3 after initiation of RRT. Competing risk endpoints were successful liberation from RRT or death without prior liberation from RRT.

**Results:**

Low pre-RRT penKid levels (penKid ≤ 89 pmol/l) at RRT initiation were associated with early and successful liberation from RRT compared to patients with high pre-RRT penKid levels (subdistribution hazard ratio (sHR) 1.83, 95%CI 1.26–2.67, *p* = 0.002, estimated 28d-cumulative incidence function (28d-CIF) of successful liberation from RRT 61% vs. 45%, *p* = 0.022). This association persisted in the landmark analysis on day 3 of RRT (sHR 1.78, 95%CI 1.17–2.71, *p* = 0.007, 28d-CIF of successful liberation from RRT 67% vs. 47%, *p* = 0.018). For both time points, no difference in the competing event of death was detected.

**Conclusions:**

In critically ill patients with RRT-dependent AKI, plasma penKid appears to be a useful biomarker for the prediction of shorter duration and successful liberation from RRT and may allow an individualized approach to guide strategies of RRT liberation in critically ill patients with RRT-dependent AKI.

*Trial registration:* The ELAIN trial was prospectively registered at the German Clinical Trial Registry (Identifier: DRKS00004367) on 28th of May 2013.

**Graphical Abstract:**

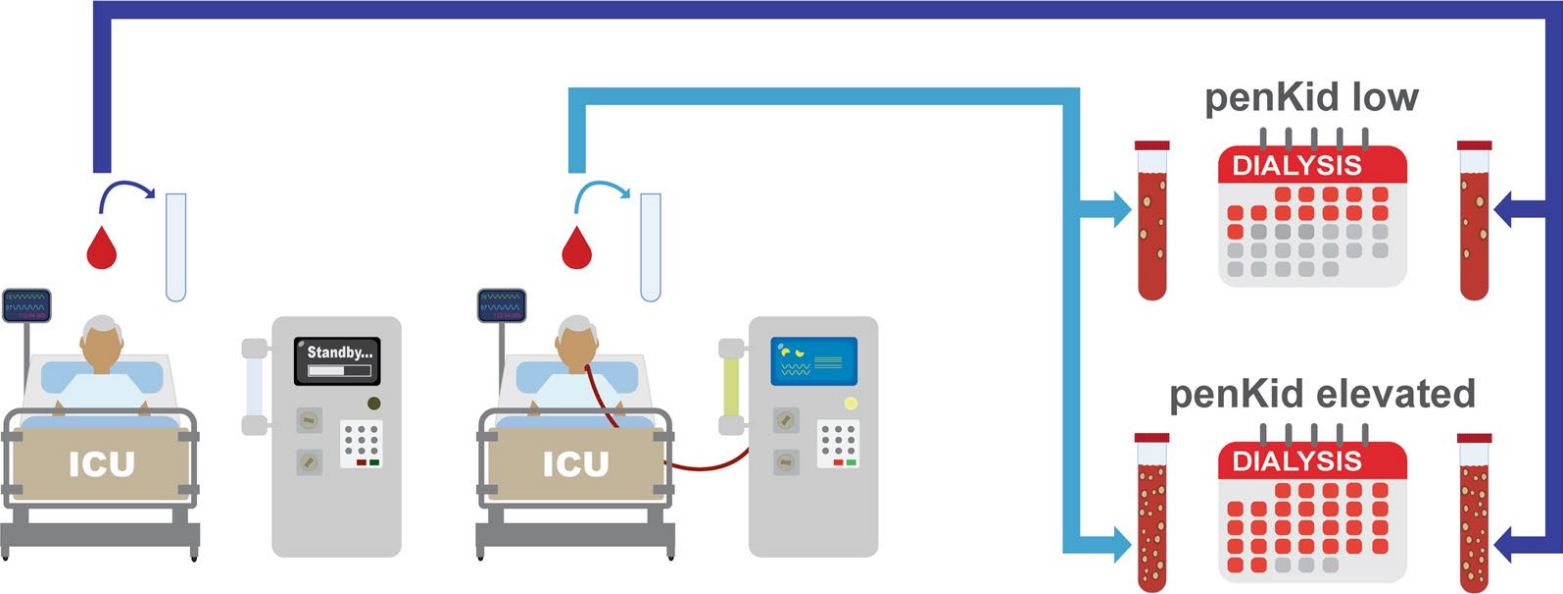

**Supplementary Information:**

The online version contains supplementary material available at 10.1186/s13054-022-04217-4.

## Introduction

Acute kidney injury (AKI) is a common and often severe complication in critically ill patients with approximately 5 to 13% of patients requiring renal replacement therapy (RRT) [1, 2]. Given the lack of specific treatment options, RRT remains pivotal to treat severe complications of AKI, such as electrolyte or acid–base dysbalances, uremia or fluid overload. RRT provides the necessary time and circumstances for the kidneys to recover and may be discontinued once adequate kidney function is restored. However, RRT carries significant risks, such as electrolyte or fluid abnormalities, hypothermia, air embolism or complications related to vascular access such as infections or bleeding. Although the causal relationship between initiation of RRT and long-term outcomes is not established, high rates of long-term sequelae and morbidity following it may limit the long-term quality of life in RRT-AKI survivors [3, 4]. Therefore, the duration of RRT must be limited to absolutely necessary periods. While several large trials have investigated the optimal timing to initiate RRT in critically ill patients with AKI, there is still a lack of high-quality evidence and tools to guide discontinuation from RRT [5–7]. Current guidelines only provide an expert-based recommendation to “discontinue RRT when it is no longer required, either because intrinsic kidney function has recovered to the point that it is inadequate to meet patient needs, or because RRT is no longer consistent with the goals of care”[8].

Improvement of urinary output is the most commonly used indicator of recovering kidney function in patients with RRT-dependent AKI [9, 10]. However, the predictive performance of urinary output remains controversial [11, 12]. Finally, urinary output thresholds and the value of biomarkers remained to be evaluated [13]. While currently being the most important predictor of successful RRT discontinuation in several studies, the exact value of urinary output remains unclear. Although several studies reported an increase in the urinary output as an important predictor of successful RRT discontinuation, the exact prognostic value of urinary output remains unclear. While a post hoc analysis of the BEST Kidney study found urinary output of more than 436 mL per day at the time of RRT cessation to be the most important predictor of successful RRT weaning [11], others reported low sensitivity and specificity of urinary output as a predictor for successful RRT liberation [12, 13]. Overall heterogeneity of cutoffs used for 24 h urinary output hamper interpretability and generalisability of these studies and few have tested this approach in prospective or randomized studies. Importantly, an increase in diuresis likely influences physician’s behavior to terminate RRT. Therefore, this may lead to bias and its prognostic performance may be overestimated.

Based on these shortcomings of currently available tools, the decision to terminate RRT still all too often employs a “one-size-fits-all approach” and is largely based on expert opinion and clinical judgment. Better tools for the assessment of individual patients are required, and biomarkers offer a unique opportunity to fill this gap. Biomarkers may provide insight into clinically hidden developments and may allow for an individual assessment of renal function recovery in patient receiving RRT. However, no such biomarker is available to identify patients with a high chance to be successfully liberated from RRT and only plasma NT-pro-BNP was identified as possibly associated with successful weaning from RRT in a study with considerable limitations [14]. Biomarkers of renal or tubular injury, such as urinary tissue inhibitor of metalloproteinase 2 and insulin-like growth factor-binding protein 7 [TIMP2]*[IGFBP7] or serum serum osteopontin (OSN) were found to be associated with renal recovery upon RRT discontinuation; however, this was not used to guide RRT discontinuation itself [15, 16]. Other kidney damage markers such as plasma neutrophil gelatinase-associated lipocain (NGAL) do not achieve adequate prognostic performance to predict RRT discontinuation [17]. This urgent need for a competent biomarker to guide liberation from RRT was also recognized in the most recent Acute Dialysis Quality Initiative (ADQI) consensus conference [18]. The statement also emphasized the need for RRT patient management to better align with the current focus on personalized medicine, calling for a “precision CRRT” approach.

Proenkephalin A 119–159 (penKid) has been investigated as a biomarker of kidney function with potential applications in patients with AKI. Interestingly, penKid is able to measure real-time glomerular filtration rate (GFR) even in non-stable settings [19] and unaffected by systemic critical illness, inflammation, age or gender [20–22]. PenKid is a more stable fragment of the endogenous opioid Enkephalin which has a potential role in the regulation of kidney function by induction of diuresis, natriuresis or by inhibiting antidiuretic hormone, and it is freely filtrated in the glomeruli [23–25]. Elevated plasma concentrations of Enkephalin (and thus penKid) reflect reduced glomerular filtration rate.

Therefore, the aim of the current study was to test the hypothesis that penKid can predict the successful liberation from RRT in critically ill patients with a RRT-dependent AKI.

## Material and methods

### Study population

This study is a post hoc analysis of the randomized clinical trial “Effect of Early versus Delayed Initiation of Renal Replacement Therapy on Mortality in Critically Ill Patients with Acute Kidney Injury” (ELAIN). Ethics approval of the ELAIN trial included storage of clinical data and samples for later exploratory post hoc and secondary analyses [5]. The ELAIN trial enrolled 231 patients, 11 of whom did not receive RRT. Of another 10 patients pre-RRT plasma samples were missing; hence, 210 patients were included in this post hoc analysis.

The ELAIN trial was a randomized, clinical two-arm trial, conducted at the University Hospital Münster in Germany between August 2013 and July 2015. The trial included adult patients with moderate AKI (KDIGO stage 2), NGAL levels > 150 ng/mL at randomization and at least one of the pre-defined additional signs of critical illness (sepsis, requirement for vasopressors or catecholamines, refractory fluid overload or non-renal organ dysfunction). At inclusion, patients were randomized to receive either early (KDIGO stage 2) or late (KDIGO stage 3 or no RRT) initiation of RRT. Patients with preexisting chronic kidney disease (eGFR < 30 mL/min), previous RRT, AKI caused by permanent occlusion or surgical lesion of the renal artery, glomerulonephritis, interstitial nephritis, vasculitis, postrenal obstruction or hemolytic uremic syndrome, thrombotic thrombocytopenic purpura, pregnancy, prior kidney transplantation, hepatorenal syndrome, AIDS with a CD4 count of < 0.05 × 10 E/L and hematologic malignancy with neutrophils of < 0.05 × 10 E/L were excluded.

In the ELAIN cohort, plasma samples were routinely collected on the day of randomization (AKI stage 2), as well as on the following days 1, 3 and 7. For this post hoc analysis, only those patients were selected for whom a penKid measurement was available within 48 h before initiation of RRT. The last penKid measurement before initiation of RRT was defined as the pre-RRT penKid measurement for each patient. Because of randomization, initiation of RRT occurred at different time points in the patient population. Consequently, the pre-RRT penKid sample is not always the measurement from the day of randomization. The established penKid cut-off value of 89 pmol/l was used to divide patients into groups of high or low penKid, accordingly [26]. Patients with penKid below the upper normal range of 89 pmol/l were assigned to the low penKid group. This cut-off is based on the manufacturer information and optimal fit of this cut-off was confirmed by optimal cut-off analysis on the dataset (Fig. 2).

### Data collection

This study used clinical data of the ELAIN trial database. Data collected in the study CRF included data about the medical history, demographic characteristics, ICU admission, AKI, as well as clinical data and pre-defined outcomes. Furthermore, plasma NGAL levels were available at the time point of study inclusion. AKI was diagnosed based on the KDIGO 2012 definition [27].

### Measurement of the biomarkers

Prior to biomarker measurement, blood samples were stored in the ELAIN trial biobank at -80 degrees Celsius after centrifugation at 5000 rpm for 5 min. Plasma samples were passively defrosted in ice water and consecutively exposed to room temperature. Biomarker measurements were performed in a blinded fashion.

Proenkephalin A 119–159 was measured in EDTA plasma samples using the immunoluminometric sphingotest® penKid® assay (SphingoTec GmbH, Hennigsdorf, Germany). Two monoclonal antibodies are directed against the middle portion of penKid and the C terminus of penKid [22]. Prior to measurement, assay calibration was performed. Samples and calibrators (50 μL) were pipetted intopolystyrene 96-well microtiter plates (Greiner Bio-One International AG, Austria). Next, labeled anti-proenkephalin A 129–144 mAb (150 μL) was added and the microtiter plates were incubated overnight for a period of 21 h at 20 °C without agitation. A washing solution (350 μL per well, five times) removes unbound tracer and the remaining chemiluminescence was measured for one second per well. PenKid levels were determined by a five-point calibration curve (25,4–2,059 pmol/l). Duplicate runs were performed for both calibrators and samples, and a coefficient of variation (CV) was required to be < 20% between duplicates. The mean value of duplicates of each sample was used for the statistical analysis.

### Outcome definitions

We determined the duration of RRT, i.e., the time from the start of RRT (day 0) to its termination, and distinguished two competing events: “successful liberation from RRT” and “death without prior liberation from RRT.” We defined the latter as death during RRT or directly (within 48 h) after RRT discontinuation to also account for palliative discontinuation of RRT. Patients who survived at least 48 h after discontinuation of RRT were classified as successfully liberated. Patients who still required RRT after day 28 were censored at day 28, because this study aimed to investigate the short- and midterm predictive value of penKid in this highly dynamic cohort of critically ill patients with RRT-AKI.

### Statistical analysis

Statistical analyses were conducted using *R* (Version R-4.1.2 for Windows) [28], and the publicly available packages *ComparisonCR, dplyr, haven, pROC, prodlim, rstatix, survival, survminer and table 1*. All analyses were conducted as exploratory analyses of hypothesis generation and were therefore not adjusted for multiple testing. All *p*-values and confidence limits were two-sided and were intended to be exploratory, not confirmatory. In this exploratory sense, *p*-values ≤ 0.05 were considered as statistically noticeable.

Baseline variables were assessed, and as applicable, frequencies, percentages, medians, quartiles, means, standard deviations and *p*-values were calculated. To compare baseline characteristics between pre-RRT penKid groups (low vs. high), Fisher’s exact test was used to compare categorical variables. Continuous variables were compared using the Mann–Whitney *U* test. Group characteristics were evaluated in the same way at the time of landmark analysis (day 3 of RRT).

To answer the first of the posed research questions, we investigated the association of pre-RRT penKid group (penKid ≤ 89 pmol/l vs. penKid > 89 pmol/l) with the time to successful liberation from RRT, controlling for death without prior liberation as a competing risk to account for differences in mortality between the two groups. For both competing outcomes (successful liberation from RRT; death without prior liberation from RRT), we estimated the cumulative incidence using the Aalen–Johansen estimator [29]. Gray's k-sample test was applied to compare the cumulative incidence of the corresponding event type between penKid groups [30]. Comparisons of the cumulative incidence functions at fixed time points were made with the methods proposed by Chen et al. using Gaynor's variance and log–log-transformed cumulative incidence functions [31]. To quantify the association between the plasma penKid group and the incidence of each competing event, we fitted two univariate Fine and Gray models, resulting in an estimate of the subdistribution hazard ratio (sHR) for each competing event [32]. In order to explain the results on a more causal level, we additionally estimated the cumulative cause-specific hazard for each competing event using the cause-specific Nelson–Aalen estimator and compared them between penKid groups using logrank tests [33]. This is helpful because cause-specific hazards represent the immediate risk for a particular event considering events of the competing type as independent censoring. In this way, the influences of the two competing events on the cumulative incidences can be analyzed separated by event. To quantify the predictive power of penKid with respect to successful liberation from RRT in a classical manner, we also generated ROC curves for the prediction of successful liberation (yes/no) by day 7 and day 28, respectively. Patients who were classified as "dead without prior liberation from RRT" up to these days as well as patients who still required RRT were grouped together as not successfully liberated for this analysis. The AUC with bootstrap 95%CIs and the sensitivity and specificity maximizing Youden’s index are reported.

To justify our choice of the penKid cut-off value, we fitted Fine and Gray models for “successful liberation from RRT” of the above type for each possible and clinically meaningful penKid cut-off value present in the data between 60 pmol/l and 120 pmol/l and plotted the associated sHR and *p*-value against the cut-off.

To investigate the predictive power of penKid also in patients during ongoing RRT (second hypothesis), we performed a landmark analysis on day 3 of RRT. For this purpose, we selected all patients who were still at risk (i.e. receiving RRT) on day 3 of RRT and defined their last available penKid measurement in the period 1–3 days after the start of RRT (day 0) as the landmark penKid value. In this way, we ensured that the landmark penKid measurement took place no more than 48 h before the landmark and already during RRT. We then repeated the statistical analyses described above with this landmark penKid value for the time interval from day 3 to day 28, again dividing the patients into two groups with low and high landmark penKid values, using the same cutoff value of 89 pmol/l as before. Event times were thus recalculated starting from the landmark time point. In order to compare both analyses (pre-RRT and landmark) visually, the same time axis was used in the figures. ROC analyses were performed for day 10 (7 days after landmark) and day 28 of RRT.

Finally, in order to investigate whether penKid may be of predictive value regarding the time point of RRT initiation (early versus late RRT initiation strategy), we divided the patient population into the two pre-RRT penKid groups and performed a separate competing risk analysis (with the same outcome variables as in the pre-RRT penKid analysis) for each penKid subgroup, now choosing the randomization group as the independent variable instead of the penKid group. This can be considered a subgroup analysis of the original analysis in the ELAIN cohort with subgroups now determined by penKid.

## Results

### Baseline characteristics

**Fig. 1:**
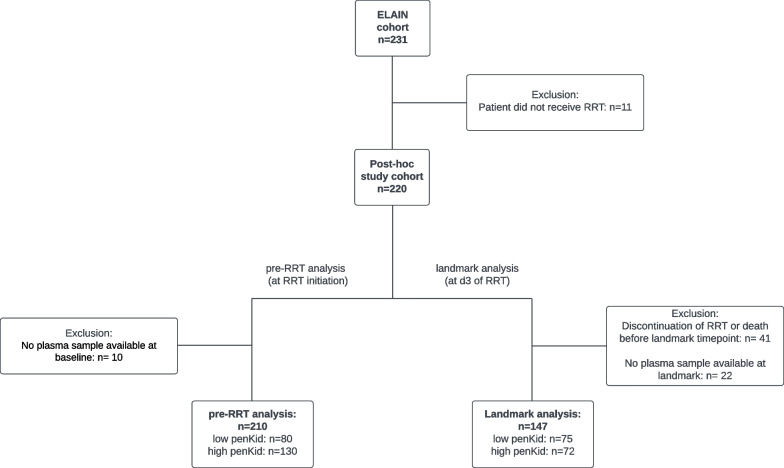
Patient flowchart for this post hoc analysis of the ELAIN trial

The ELAIN trial included 231 patients, 210 of whom were included in this post hoc study. The study workflow is shown in the study flowchart (Fig. 1). Baseline characteristics of the study cohort are shown in Table 1. Baseline characteristics did not differ noticeably between groups except for estimated GFR (Median [Q1-Q3] eGFR: high penKid 35 [28–44] vs. low penKid 46 [32–59] ml/min/1.73m2, *p* < 0.001). Distribution of pre-RRT penKid values are displayed in Additional file 1: Figure S1.

**Table 1:** Patient characteristics at baseline; all variables were collected at the time of randomization except for estimated GFR, which was measured at the time of pre-RRT penKid measurement

Variable	Total (n = 210)	Low penKid (≤ 89 pmol/l) (n = 80)	High penKid (> 89 pmol/l) (n = 130)	*p*-value
Sex				
Male	132 (62.9%)	56 (70.0%)	76 (58.5%)	0.107^a^
Female	78 (37.1%)	24 (30.0%)	54 (41.5%)	
Age (years)				
Median (Q1, Q3)	70.0 (59.0, 76.0)	69.0 (57.8, 74.3)	71.0 (60.0, 77.0)	0.148^b^
Mean (SD)	66.7 (13.3)	65.2 (13.6)	67.7 (13.0)	
Creatinine (mg/dl)				
Median (Q1, Q3)	1.10 (0.800, 1.40)	1.00 (0.800, 1.20)	1.10 (0.800, 1.40)	0.195^b^
Mean (SD)	1.13 (0.406)	1.08 (0.368)	1.17 (0.426)	
Missing	4 (1.9%)	1 (1.3%)	3 (2.3%)	
Estimated GFR (ml/min/1.73m^2^)				
Median (Q1, Q3)	37.5 (30.0, 48.0)	44.5 (32.3, 59.0)	35.0 (28.0, 44.0)	< 0.001^b^
Mean (SD)	44.3 (29.2)	54.3 (40.7)	38.1 (15.9)	
Missing	8 (3.8%)	2 (2.5%)	6 (4.6%)	
SOFA score				
Median (Q1, Q3)	14.0 (12.0, 16.0)	14.0 (13.0, 16.0)	14.0 (12.0, 16.8)	0.530^b^
Mean (SD)	14.1 (3.06)	14.0 (2.92)	14.2 (3.16)	
APACHE II				
Median (Q1, Q3)	25.0 (20.0, 32.0)	25.0 (21.0, 33.0)	25.0 (20.0, 32.0)	0.411^b^
Mean (SD)	26.3 (7.69)	26.9 (7.02)	25.9 (8.09)	
Hypertension				
Yes	173 (82.4%)	67 (83.8%)	106 (81.5%)	0.714^a^
No	37 (17.6%)	13 (16.3%)	24 (18.5%)	
Diabetes				
Yes	37 (17.6%)	12 (15.0%)	25 (19.2%)	0.463^a^
No	173 (82.4%)	68 (85.0%)	105 (80.8%)	
Chronic obstructive pulmonary disease (COPD)				
Yes	37 (17.6%)	18 (22.5%)	19 (14.6%)	0.191^a^
No	173 (82.4%)	62 (77.5%)	111 (85.4%)	
Vasopressors				
Yes	187 (89.0%)	71 (88.8%)	116 (89.2%)	1.000^a^
No	23 (11.0%)	9 (11.3%)	14 (10.8%)	
Kidney biomarker NGAL (ng/ml)				
Median (Q1, Q3)	555 (368, 879)	603 (355, 1010)	529 (372, 827)	0.640^b^
Mean (SD)	657 (360)	681 (380)	642 (347)	

^a^Fisher’s exact test comparing the low and high pre-RRT penKid group.

^b^Mann–Whitney *U* test comparing the low and high pre-RRT penKid group

*APACHE II*, Acute Physiology and Chronic Health Evaluation II, *GFR*, Glomerular Filtration Rate; *NGAL*, Neutrophil gelatinase-associated lipocalin; *SD*, Standard Deviation; *SOFA*, Sequential Organ Failure Assessment

### Sensitivity analysis of penKid cutoff

**Fig. 2:**
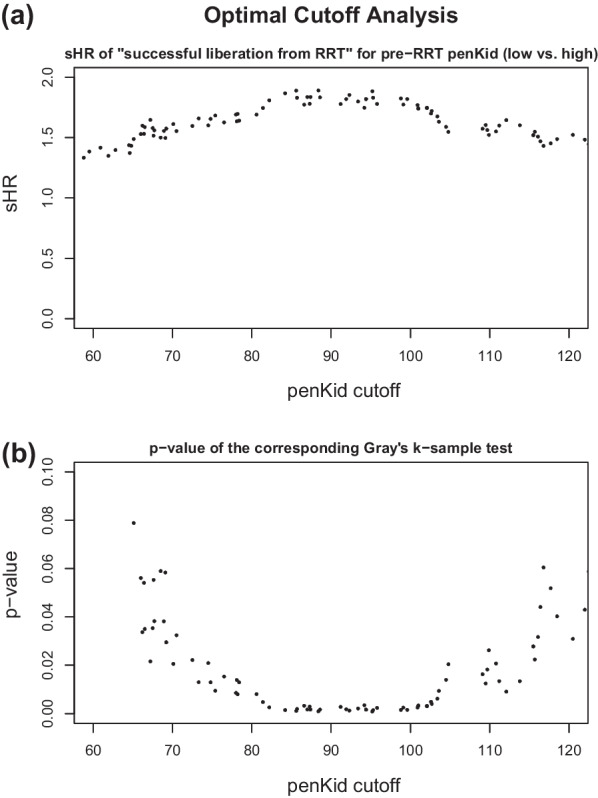
**a** Estimated sHR for “successful liberation from RRT” between pre-RRT penKid groups (low vs. high) as a function of all possible penKid cutoff values between 60 pmol/l and 120 pmol/l present in the data. **b** *p*-value of the corresponding Gray’s k-sample test (H_0_: sHR = 1) as a function of all possible penKid cutoff values between 60 pmol/l and 120 pmol/l present in the data

The association of the pre-RRT penKid group with successful liberation from RRT is strongest for cutoff values between 85 pmol/l and 100 pmol/l (Fig. 2). The upper normal range of 89 pmol/l is therefore reasonably chosen in this dataset.

### RRT duration pre-RRT sample analysis

In the first 28 days after initiation of continuous RRT, RRT was successfully discontinued in 107/210 patients (28d-CIF 51%) of the study cohort. Successful liberation from RRT occurred considerably more frequently in the low penKid group (49/80, 28d-CIF 61%) compared to the high penKid group (58/130, 28d-CIF 45%), *p* = 0.022. In contrast, no mortality difference was detectable between the low and the high penKid group. Overall, 65/210 patients (28d-CIF 31%) died within 48 h after discontinuation from RRT during this 28-day period. This affected 23/80 patients (28d-CIF 29%) with low pre-RRT penKid and 42/130 (28d-CIF 32%) with high pre-RRT penKid.

**Fig. 3:**
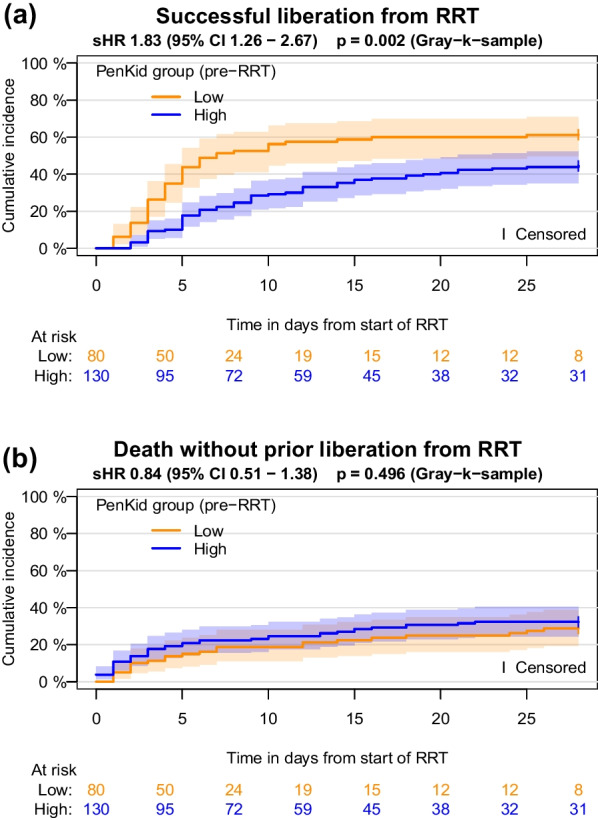
Estimated cumulative incidence functions of successful liberation from RRT **a** and death without prior liberation from RRT **b** with log–log-transformed pointwise 95% confidence intervals

Estimated cumulative incidence functions demonstrate that patients with a low penKid value (≤ 89 pmol/l) tended to require less time on RRT to renal recovery compared to patients with a high penKid value (> 89 pmol/l) (Fig. 3a). The associated univariate Fine & Gray model yields a sHR of 1.83 (95% CI 1.26–2.67, *p* = 0.002) for low versus high penKid group. Since the graph shows that the assumption of proportional subdistribution hazards is violated, this estimate is to be interpreted as a time average. In terms of mortality, no remarkable difference is observed (Fig. 3b).

Estimated cumulative cause-specific hazards demonstrate that the difference in the incidence of successful liberation from RRT is not due to a reduced mortality hazard in the low penKid group, but can in fact be explained by an increased hazard for successful liberation from RRT in this group during the first week of RRT. Additional file 2: Figure S2a shows that the cause-specific hazard for successful liberation from RRT was markedly higher in the low penKid group than in the high penKid group during this period (cause-specific hazard ratio (cHR) 1.97, 95%CI 1.35–2.89, *p* < 0.001). On the seventh day, 41/80 of the low penKid patients (7d-CIF 51%), but only 29/130 of the high penKid patients (7d-CIF 22%) were successfully liberated from RRT, *p* < 0.001. From day 7 onwards, this difference gradually diminished. Together with a slightly increased mortality hazard in the low penKid group during the second half of the 28-day observation period (Additional file 2: Figure S2b) this explains why the cumulative incidence functions for successful liberation approach each other again toward the end of the 28-day observation period. Results of the ROC analysis are provided in Supplement (Additional files 5, 6: Figures S5, S6).

### Landmark analysis

Within the first two days (day 0 to day 2) of RRT, 15 of the 210 patients included in this post hoc analysis were successfully liberated. In 26 patients, RRT was discontinued for palliative reasons; they died within 48 h after discontinuation of RRT. 169 were still at risk at the start of day 3 of RRT. In 22 of these patients, no landmark penKid value could be determined due to missing measurements and they were therefore excluded from the landmark analysis. This affected 9 patients in the low pre-RRT penKid group and 13 patients in the high pre-RRT penKid group. Thus, a total of 147 patients were available for examination in the landmark analysis. Landmark characteristics of the study cohort are shown in Additional file 9: Table S1 and the characteristics did not differ noticeably between groups except for creatinine and estimated GFR (Median [Q1-Q3] creatinine: high penKid 1.2 [0.9–1.6] versus low penKid 1.0 [0.8–1.3] mg/dl, *p* = 0.010; Median [Q1-Q3] eGFR: high penKid 43 [35–50] versus low penKid 66 [55–113] ml/min/1.73 m^2^, *p* < 0.001).

**Fig. 4:**
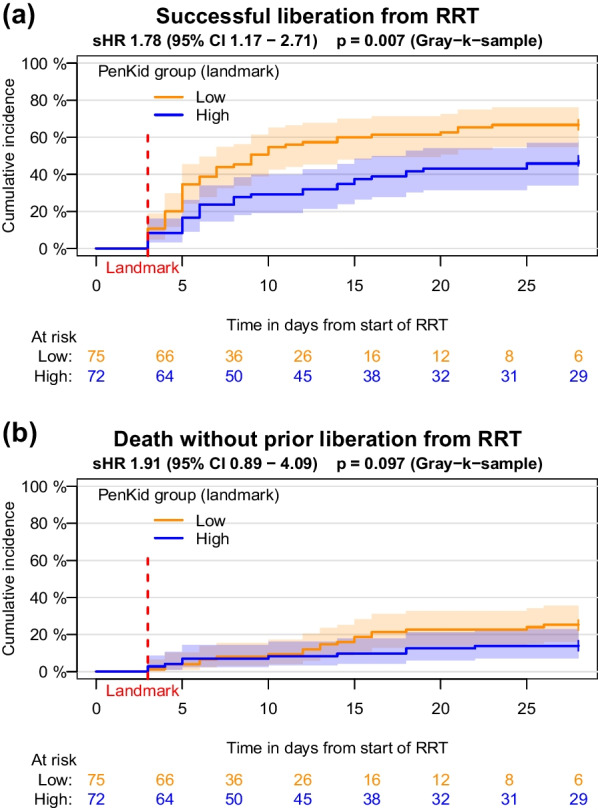
Estimated cumulative incidence functions of successful liberation from RRT **a** and death without prior liberation from RRT **b** with log–log-transformed pointwise 95% confidence intervals, starting at the landmark on day 3 and including all patients still receiving RRT on the third day

The results of the landmark analysis were in line with the pre-RRT sample analysis. Within the time interval of the landmark analysis (day 3—day 28), RRT was successfully discontinued in 84/147 patients (28d-CIF 57%) of the study cohort. Successful liberation from RRT occurred considerably more frequently in the low penKid group (50/75, 28d-CIF 67%) compared to the high penKid group (34/72, 28d-CIF 47%), *p* = 0.018. The associated univariate Fine & Gray model yields a sHR of 1.78 (95% CI 1.17–2.71, *p* = 0.007) (Fig. 4a). Mortality in the low penKid group in the second half of the 28-day observation period appeared more pronounced than in the pre-RRT sample analysis, but not statistically noticeable; 29/147 patients (28d-CIF 20%) died within 48 h after discontinuation from RRT between day 3 and day 28, 19/75 patients (28d-CIF 25%) with low and 10/72 (28d-CIF 14%) with high landmark penKid value (Fig. 4b).

The cause-specific hazards also paint a similar picture as in the pre-RRT sample analysis, with the exception of two differences. First, the cause-specific hazard for successful liberation in the low landmark penKid group seems to be constantly higher than in the high landmark penKid group (cHR 2.09, 95% CI 1.34–3.26, *p* = 0.001); even after the first week, the hazards seem to converge only slightly (Additional file 3: Figure S3a). Second, the increase in mortality hazard in the low penKid group in the second half of the observation period is much more pronounced compared to the pre-RRT analysis (Additional file 3: Figure S3b). The difference between both groups is statistically noticeable (cHR 3.39, 95% CI 1.55–7.42, *p* = 0.002). Results of the ROC analysis are provided in Supplement (Additional file 7, 8: Figures S7, S8).

### Early versus late RRT initiation group analysis

This analysis includes all 210 patients of the study. The estimated cumulative incidence functions separated by pre-RRT penKid group (low/high) and randomization group (early or delayed initiation of RRT) are presented in Additional file 4: Figure S4. For patients with low pre-RRT penKid, no difference in rates of successful liberation from RRT was observable between randomization groups. However, among patients with high pre-RRT penKid, patients in the early randomization group were successfully liberated from RRT considerably earlier and more frequently (sHR 1.69, 95%CI 1.02–2.79, *p* = 0.042). No considerable difference in terms of death without prior liberation from RRT was observable.

## Discussion

Low penKid values (penKid ≤ 89 pmol/l) before RRT initiation were associated with shorter duration of RRT and higher incidence of successful liberation from RRT, especially in the first two weeks after RRT initiation. Competing risk analysis revealed that this higher incidence of successful liberation from RRT was due to an increased hazard of successful RRT liberation and that there was no noticeable difference in the competing event of death without prior liberation from RRT. Second, the early versus late initiation of RRT group analysis suggested that patients with high pre-RRT penKid had higher rates and earlier successful liberation from RRT if randomized to the “early RRT initiation” group, compared to patients in the “delayed RRT initiation” group. Finally, the landmark analysis performed in patients on RRT demonstrated that low penKid levels during RRT were also associated with early and successful liberation from RRT, suggesting that penKid may be a competent marker of kidney function even during ongoing RRT. Hence, it may reflect kidney function “under” RRT.

Interestingly, there was an increase in mortality hazard in the low penKid group in the second half of the observation period. However, since the mortality hazard only increases in the second half, we assume that the increase is not due to the low penKid values but due to unobserved comorbidities or critical illness. We hypothesize that in the low penKid group, a natural patient selection occurs during the first two weeks: the patients primarily suffering from renal dysfunction can mostly be successfully liberated from RRT within the first two weeks, as their renal function recovers well (low penKid values). After two weeks, those patients remain who have other comorbidities or persisting critical illness and therefore could not be safely liberated from RRT within the first two weeks. In this higher than average comorbid collective, the mortality hazard is then increased in the second half of the observation period. In the high penKid group, this selection does not occur to the same extent, since the generally poorer renal function means that even the non-comorbid patients are less likely to be successfully liberated within the first 2 weeks and the hazard for mortality is similarly high throughout the observation period.

Our findings are in line with the pathophysiological basis of our hypothesis that low penKid reflects preserved underlying kidney function in patients already meeting KDIGO AKI criteria based on the functional biomarkers serum creatinine and oliguria. Estimated GFR was inversely correlated with penKid values, both at pre-RRT and to some extent at landmark analysis of patient cohort characteristics, affected by falsely elevated creatinine-based GFR values during ongoing RRT. This is consistent with previously published data showing a strong inverse correlation of penKid concentrations with measured GFR determined by iohexol plasma clearance [34].

PenKid is a promising novel tool on the way to make precision medicine a reality for critically ill patients with AKI. To our knowledge, this is the first study that investigates penKid during RRT and for the prediction of early and successful liberation from RRT. Results of our landmark analysis during RRT suggest that penKid may be able to assess underlying kidney function in patients receiving RRT. This could support clinicians to identify patients with adequate recovery of kidney function during RRT, suitable for liberation. Moving forward, such an application of penKid may allow to protect patients from the risks of RRT by shortening unnecessary days on RRT and also support allocation of sparse intensive care resources, thereby helping to reduce costs of unnecessarily long periods of RRT or unsuccessful termination with subsequent need for reinitiation of RRT [35]. Plasma concentrations of penKid in our cohort are in line with data of previous published studies, both in cohorts of critically ill patients generally, cardiac surgery patients with AKI and in critically ill patients with sepsis and AKI [36, 37]. Given the molecular weight of PenKid of 4.5kD, it is likely dialysable. As a prohormone fragment of the peptide hormone enkephalin, penKid may be constantly and rapidly produced in the body which may explain the constant level, assuming a balance between filtration and production.

As an increase in diuresis is commonly used in our practice to decide to terminate RRT and given the retrospective nature of the analysis, we could not investigate the true prognostic performance of this marker in our cohort. Other standard methods of kidney function assessment, such as measurement of serum creatinine, are distorted during RRT and therefore not utilizable. Serum creatinine may be used to monitor dialysis dose and intensity, but is not suitable to estimate underlying kidney function. Alternative methods, such as measurement of urinary creatinine or urinary urea excretion have been suggested, as well as testing renal response to a diuretic stimulus, such as loop diuretics [12, 38]. However, these methods also face important limitations and currently there is a lack of high-quality evidence for the implementation of these methods. Novel biomarkers have been suggested to fill this clinical gap, and small studies investigating the use of NGAL to predict successful liberation from RRT reported promising results for NGAL prior to RRT discontinuation; however, in other studies, NGAL levels were found not to be significantly associated with successful weaning from RRT [39, 40]. Our results show that NGAL concentrations were very comparable in the low/high PenKid group, illustrating that the predictive value of NGAL is limited. Other biomarkers of kidney damage, such as [TIMP2]*[IGFBP7] or NGAL may be able to identify and quantify kidney stress or damage early on in the course of AKI, but are likely of limited value for peri-RRT assessment of kidney recovery. Other markers such as urinary C–C motif chemokine ligand 14 (CCL14) have recently been suggested as potent markers of renal non-recovery; however, they are yet to be investigated for their clinical utility for guiding RRT therapy [41]. While CCL14 is a promising marker to identify patients at high risk for persistent renal impairment, it has limited ability to guide RRT liberation [42, 43]. The ability of penKid to dynamically assess kidney function is an advantage over these markers in the RRT-AKI setting to guide the decision to discontinue RRT. Whether a combination of CCL14 and penKid may have complementary effects could be a potential question for following studies. This summary of conflicting evidence underlines the lack of high-quality evidence supporting the currently suggested STOP criteria and how a biomarker could support this approach.

Our study has several limitations, which must be acknowledged. First, this is an observational post hoc analysis of exploratory nature. Therefore, results must be interpreted as hypothesis-generating. Given the research questions of this analysis were postulated after trial completion, the data and time points of biological samples from the original trial were not always optimally designed and available to answer the novel questions at hand. Concretely, this required us to define the outcome variable (liberation from RRT) retrospectively. Furthermore, plasma samples were collected only at randomization, day 1, 3 and 7, but were not available daily until RRT discontinuation, which is why the association of penKid with successful liberation from RRT could not be examined in more detail over time. It appears plausible that serial penKid samples taken, so also closer to the actual moment of termination of RRT, may increase its predictive value. Second, the ELAIN trial was a single-center study at a university hospital in Germany, potentially limiting generalizability of the results. Third, the ELAIN trial mainly included cardiac surgery patients, potentially limiting the generalizability of results to other cohorts. Finally, liberation from RRT was not guided by the study protocol, but individually managed by the treating physicians in the ICU. However, physicians in this single-center study followed the same standard operating procedures as part of local practice. Therefore, similar strategies and selection for RRT liberation can be assumed in both groups.

This work provides a basis for further evaluation of penKid in prospective studies. First, our findings warrant external validation and multicenter validation, which is currently ongoing. If successfully validated, a following randomized controlled trial could investigate whether a penKid-guided strategy of liberation from RRT improves outcomes and rates of early and successful RRT liberation. Second, our data (randomization group analysis) imply that pre-RRT penKid may be a valuable biomarker for the selection of a subgroup of patients with potential benefit from strategies to initiate RRT early. Given the heterogeneity and conflicting results of previous RCTs investigating early versus delayed strategies of RRT initiation, a precision medicine approach of biomarker enrichment may improve patient selection for such trials and clinical practice. PenKid may be a suitable candidate for such patient selection and this certainly warrants further investigation.

## Conclusion

In critically ill patients with RRT-dependent AKI, low plasma penKid levels directly before RRT initiation predict successful and early liberation from RRT. This association was still present in a landmark analysis when penKid was measured in patients during ongoing RRT. This indicates that penKid may be a potent biomarker to assess underlying kidney function in critically ill patients with AKI even during ongoing RRT. Finally, penKid at the time point of AKI diagnosis may be a useful tool to identify patients who may benefit from early RRT initiation and could support enrichment strategies of clinical trials investigating early versus late RRT initiation strategies. Moving forward, penKid may be a competent biomarker to support early risk stratification and individualization of treatment pathways for critically ill patients with RRT-dependent AKI. Our findings now require investigation in further prospective studies.

## Supplementary Information

**Additional file 1.** **Figure S1**: (a) Distribution of pre-RRT penKid values in the study cohort. (b) Boxplots are separated by pre-RRT penKid group (low: ≤89 pmol/l, high: >89 pmol/l).

**Additional file 2**. **Figure S2**: Estimated cumulative cause-specific hazard of successful liberation from RRT (a) and death without prior liberation from RRT (b) with log-transformed pointwise 95% confidence intervals.

**Additional file 3**. **Figure S3**: Estimated cumulative cause-specific hazard of successful liberation from RRT (a) and death without prior liberation from RRT (b) with log-transformed pointwise 95% confidence intervals, starting at the landmark on day 3 and including all patients still receiving RRT on the third day.

**Additional file 4**. **Figure S4**: Estimated cumulative incidence functions of successful liberation from RRT (a) and death without prior liberation from RRT (b) separated by pre-RRT penKid group and randomization group.

**Additional file 5**. **Figure S5**: ROC curve showing the prediction of successful liberation from RRT by day 7 based on the pre-RRT penKid value. The penKid threshold maximizing Youden’s index is 100.25 pmol/l yielding a sensitivity of 66% and a specificity of 66% (orange dot).

**Additional file 6**. **Figure S6**: ROC curve showing the prediction of successful liberation from RRT by day 28 based on the pre-RRT penKid value. The penKid threshold maximizing Youden’s index is 95.25 pmol/l yielding a sensitivity of 50% and a specificity of 67% (orange dot).

**Additional file 7**. **Figure S7**: ROC curve showing the prediction of successful liberation from RRT by day 10 (7 days after landmark) based on the landmark penKid value. The penKid threshold maximizing Youden’s index is 77.1 pmol/l yielding a sensitivity of 60% and a specificity of 71% (orange dot).

**Additional file 8**. **Figure S8**: ROC curve showing the prediction of successful liberation from RRT by day 28 based on the landmark penKid value. The penKid threshold maximizing Youden’s index is 74.9 pmol/l yielding a sensitivity of 52% and a specificity of 75% (orange dot).

**Additional file 9**. **Table S1**: Patient characteristics of landmark patients, i.e., patients still receiving RRT at day 3. All variables were collected at the time of randomization except for estimated GFR, which was measured at the time of landmark penKid measurement. aFisher’s exact test comparing the low and high landmark penKid group. bMann–Whitney U test comparing the low and high landmark penKid group. Abbreviations: APACHE II, Acute Physiology and Chronic Health Evaluation II, GFR, Glomerular Filtration Rate; NGAL, Neutrophil gelatinase-associated lipocalin; SD, Standard Deviation; SOFA, Sequential Organ Failure Assessment

## Data Availability

Statistical results are available from the authors upon request. The trial was registered prior to study start at the German Clinical Trial Registry, Study Identifier: DRKS00004367.

## Abbreviations

AKI: Acute kidney injury
AUC: Area under the curve
cHR: Cause-specific hazard ratio
CI: Confidence interval
CRF: Case report form
ELAIN: Effect of early versus delayed initiation of renal replacement therapy on mortality in critically Ill patients with acute kidney injury trial
GFR: Glomerular filtration rate
ICU: Intensive care unit
OR: Odds ratio
penKid: Proenkephalin A 119–159
(C)RRT: (Continuous) Renal replacement therapy
sHR: Subdistributional hazard ratio
28d-CIF: Estimated cumulative incidence function at day 28
