# Supplementary figures and images for: Proenkephalin A 119–159 predicts early and successful liberation from renal replacement therapy in critically ill patients with acute kidney injury: a post hoc analysis of the ELAIN trial
Source: Crit Care. 2022 Oct 31;26:333. doi: 10.1186/s13054-022-04217-4 (PMC9624047; doi:10.1186/s13054-022-04217-4)

**(a)**

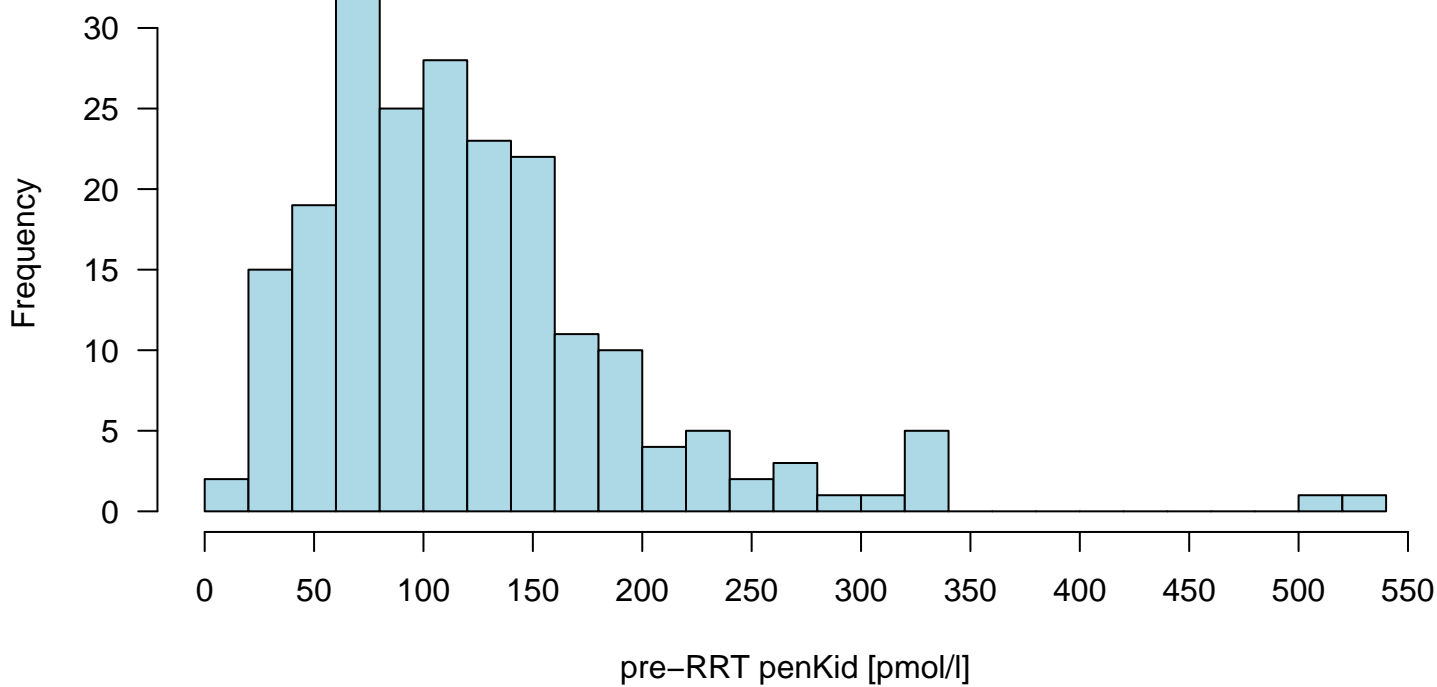

**(b)**

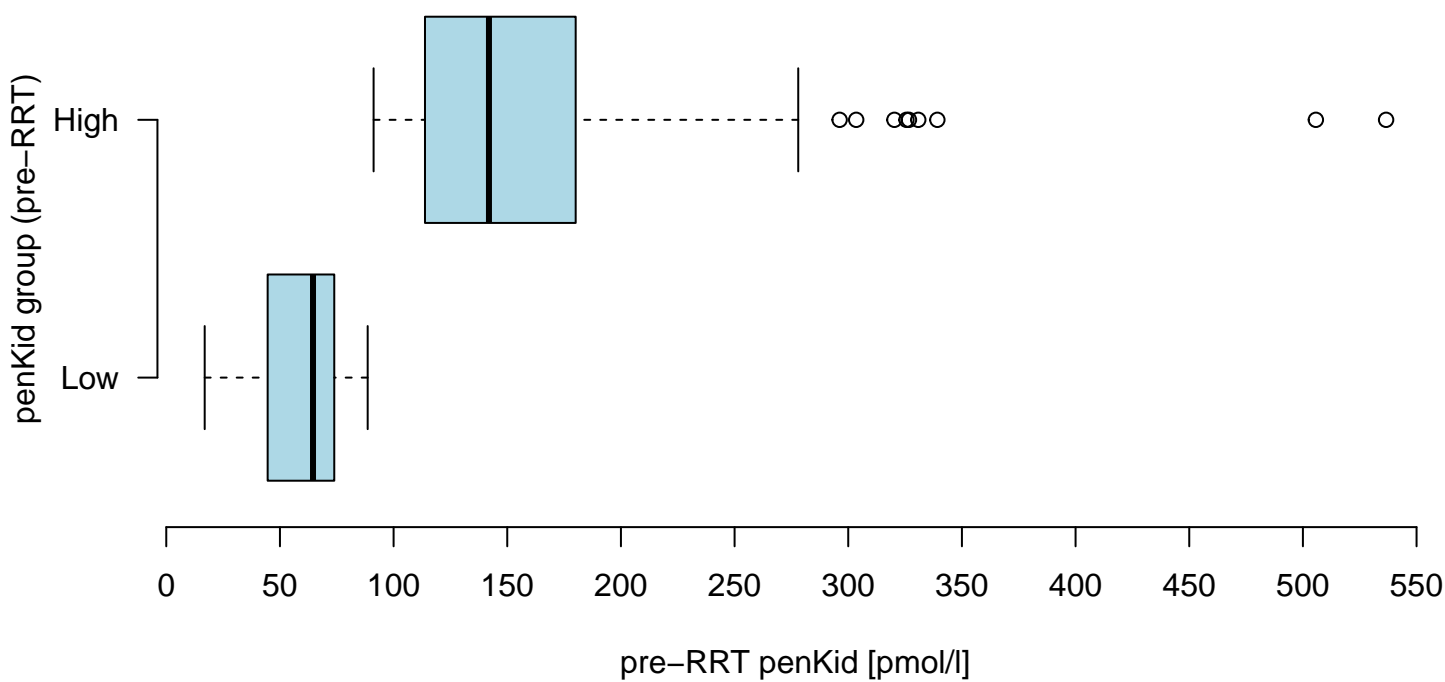

Supplement: Supplementary file 1 — Additional file 1. Figure S1: (a) Distribution of pre-RRT penKid values in the study cohort. (b) Boxplots are separated by pre-RRT penKid group (low: ≤89 pmol/l, high: >89 pmol/l). [file 13054_2022_4217_MOESM1_ESM.pdf]

(a)

## Successful liberation from RRT

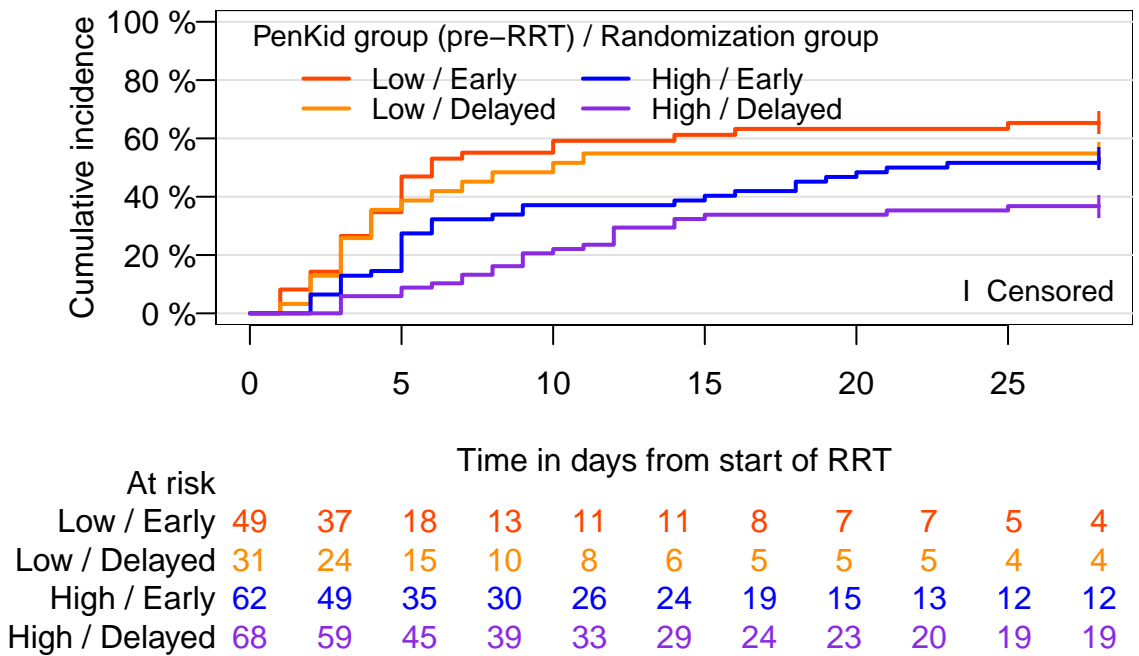

(b)

## Death without prior liberation from RRT

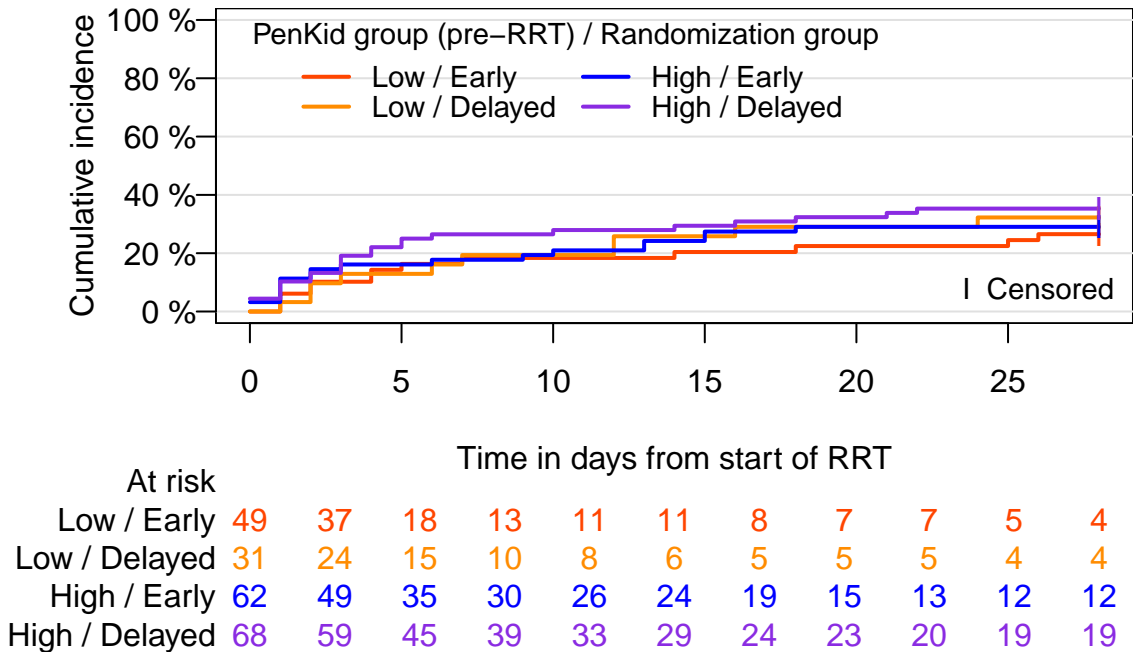

Supplement: Supplementary file 4 — Additional file 4. Figure S4: Estimated cumulative incidence functions of successful liberation from RRT (a) and death without prior liberation from RRT (b) separated by pre-RRT penKid group and randomization group. [file 13054_2022_4217_MOESM4_ESM.pdf]

**ROC Curve "Successful liberation from RRT until day 7"**

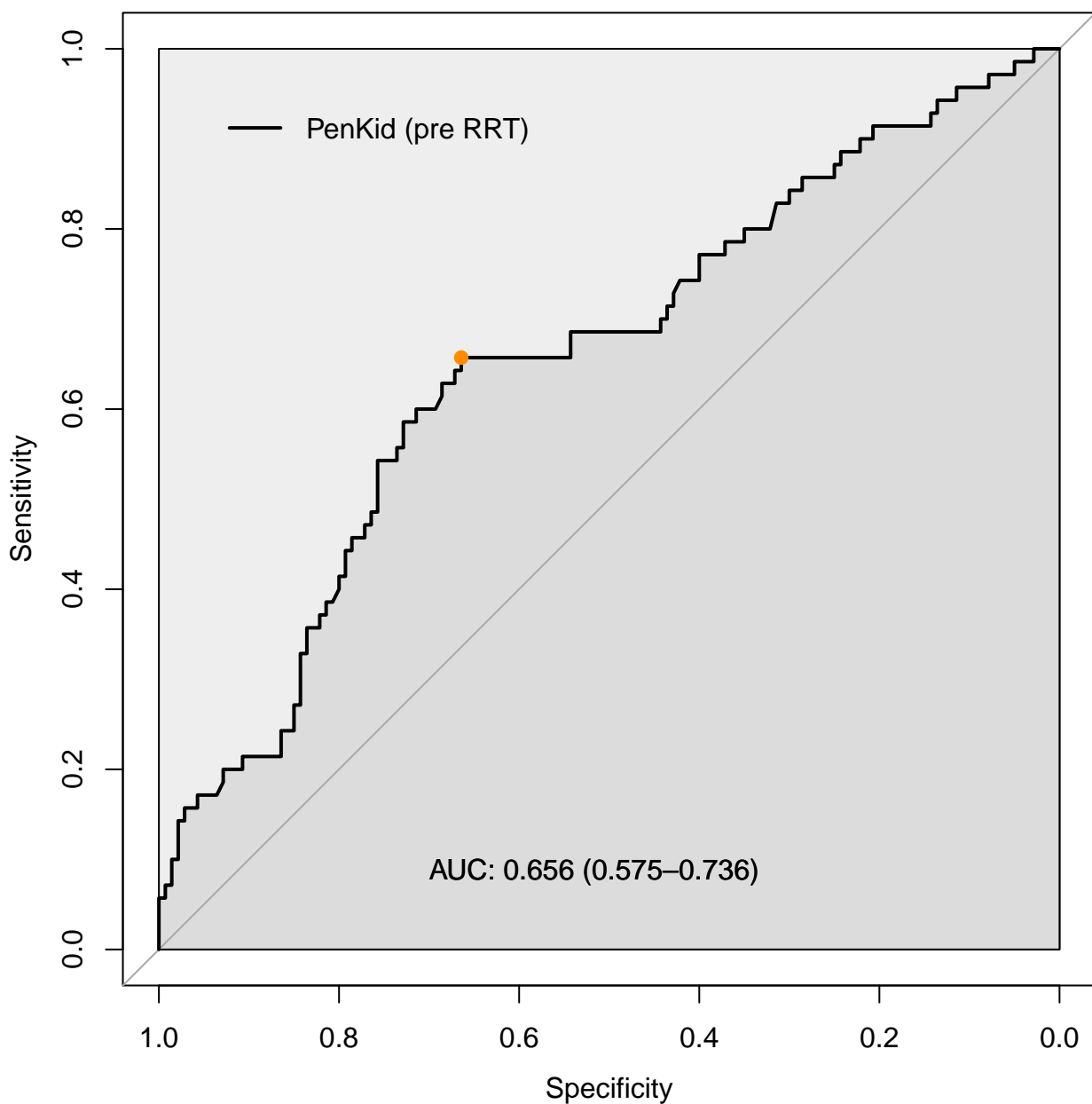

Supplement: Supplementary file 5 — Additional file 5. Figure S5: ROC curve showing the prediction of successful liberation from RRT by day 7 based on the pre-RRT penKid value. The penKid threshold maximizing Youden’s index is 100.25 pmol/l yielding a sensitivity of 66% and a specificity of 66% (orange dot). [file 13054_2022_4217_MOESM5_ESM.pdf]

**ROC Curve "Successful liberation from RRT until day 28"**

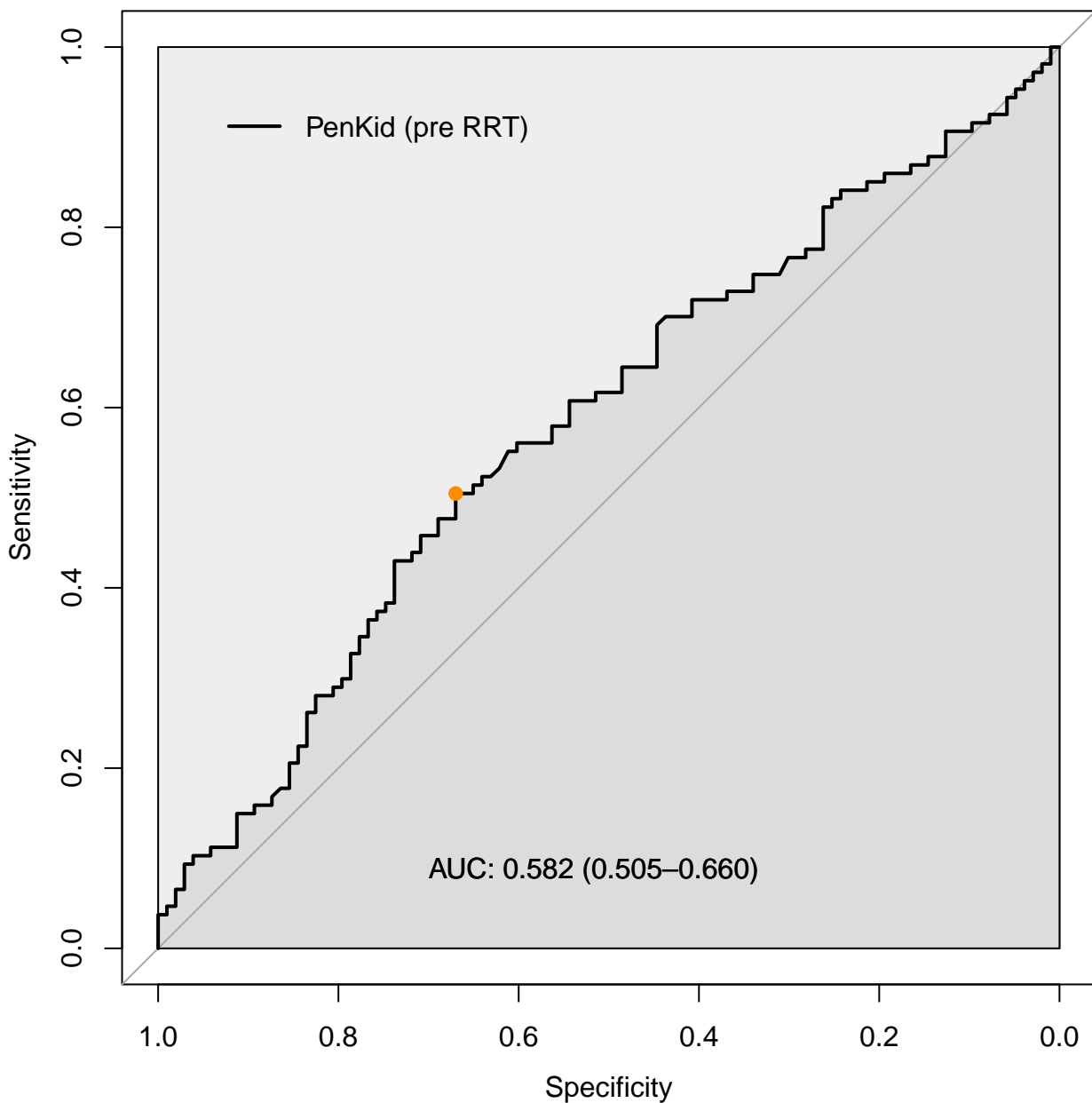

Supplement: Supplementary file 6 — Additional file 6. Figure S6: ROC curve showing the prediction of successful liberation from RRT by day 28 based on the pre-RRT penKid value. The penKid threshold maximizing Youden’s index is 95.25 pmol/l yielding a sensitivity of 50% and a specificity of 67% (orange dot). [file 13054_2022_4217_MOESM6_ESM.pdf]

**ROC Curve "Successful liberation from RRT until day 10"**

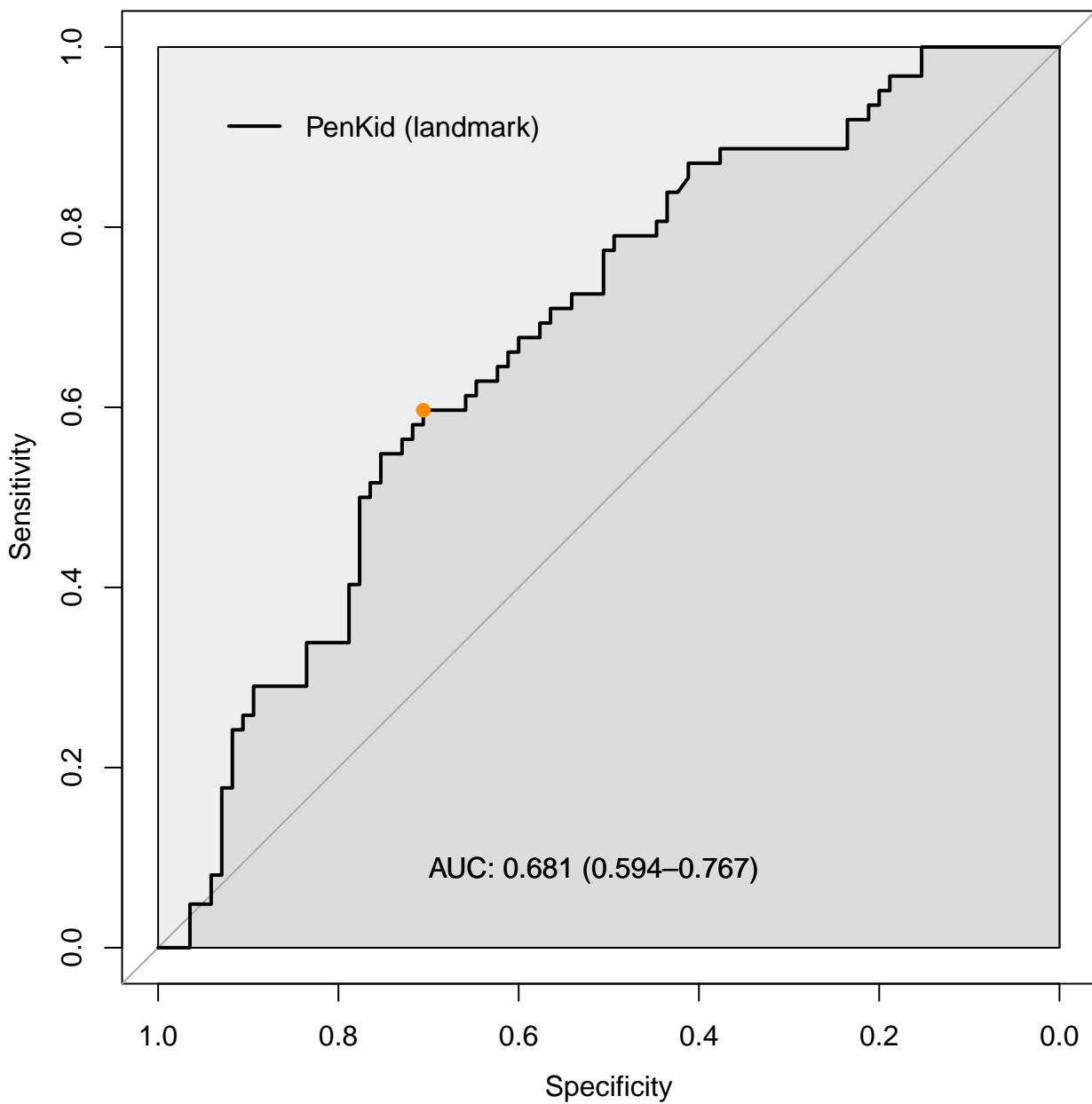

Supplement: Supplementary file 7 — Additional file 7. Figure S7: ROC curve showing the prediction of successful liberation from RRT by day 10 (7 days after landmark) based on the landmark penKid value. The penKid threshold maximizing Youden’s index is 77.1 pmol/l yielding a sensitivity of 60% and a specificity of 71% (orange dot). [file 13054_2022_4217_MOESM7_ESM.pdf]

**ROC Curve "Successful liberation from RRT until day 28"**

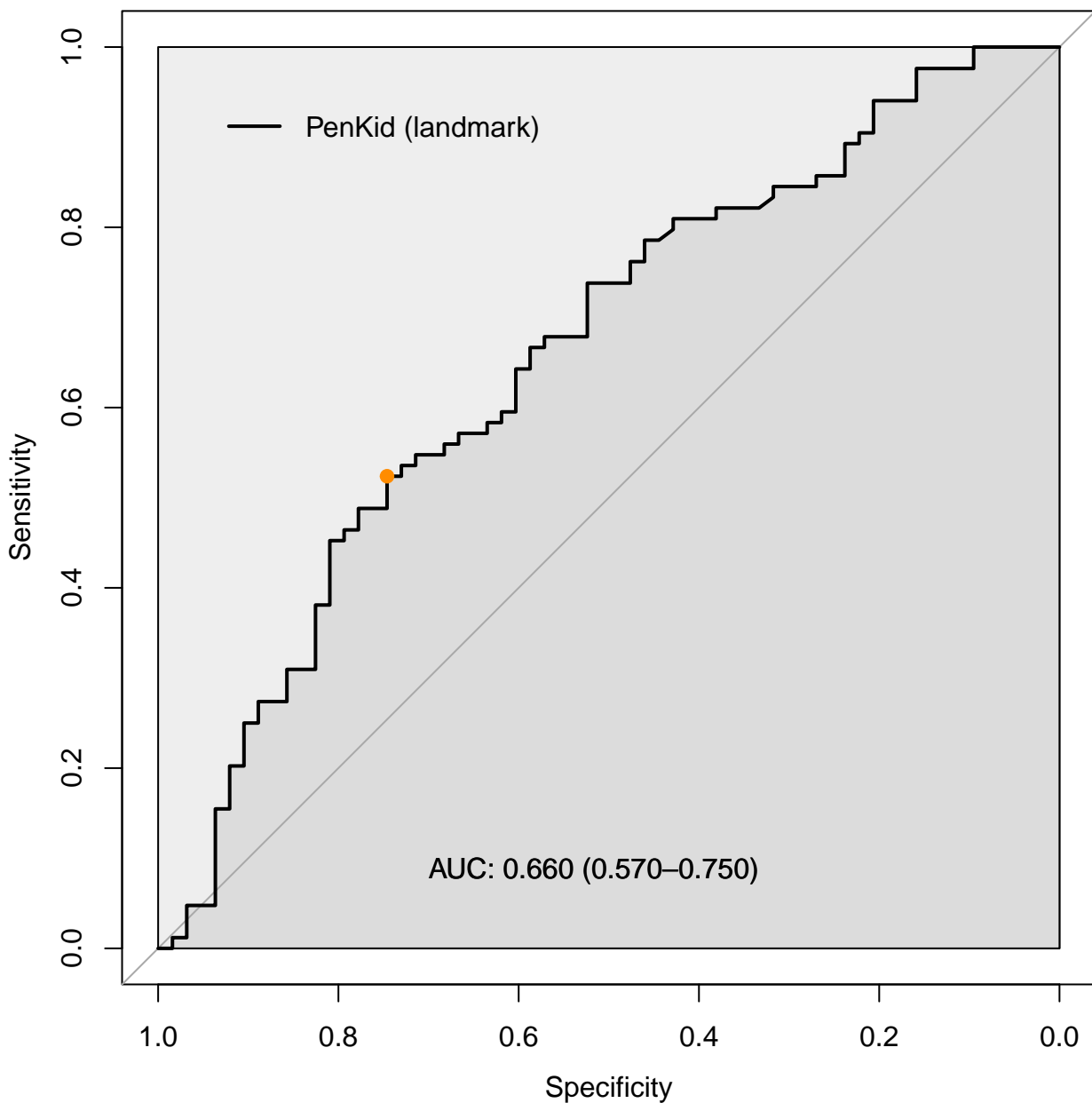

Supplement: Supplementary file 8 — Additional file 8. Figure S8: ROC curve showing the prediction of successful liberation from RRT by day 28 based on the landmark penKid value. The penKid threshold maximizing Youden’s index is 74.9 pmol/l yielding a sensitivity of 52% and a specificity of 75% (orange dot). [file 13054_2022_4217_MOESM8_ESM.pdf]
