# Supplementary material for: Proenkephalin A 119–159 predicts early and successful liberation from renal replacement therapy in critically ill patients with acute kidney injury: a post hoc analysis of the ELAIN trial
Source: Crit Care. 2022 Oct 31;26:333. doi: 10.1186/s13054-022-04217-4 (PMC9624047; doi:10.1186/s13054-022-04217-4)
Supplement: Supplementary file 2 — Additional file 2. Figure S2: Estimated cumulative cause-specific hazard of successful liberation from RRT (a) and death without prior liberation from RRT (b) with log-transformed pointwise 95% confidence intervals. [file 13054_2022_4217_MOESM2_ESM.pdf]

(a)

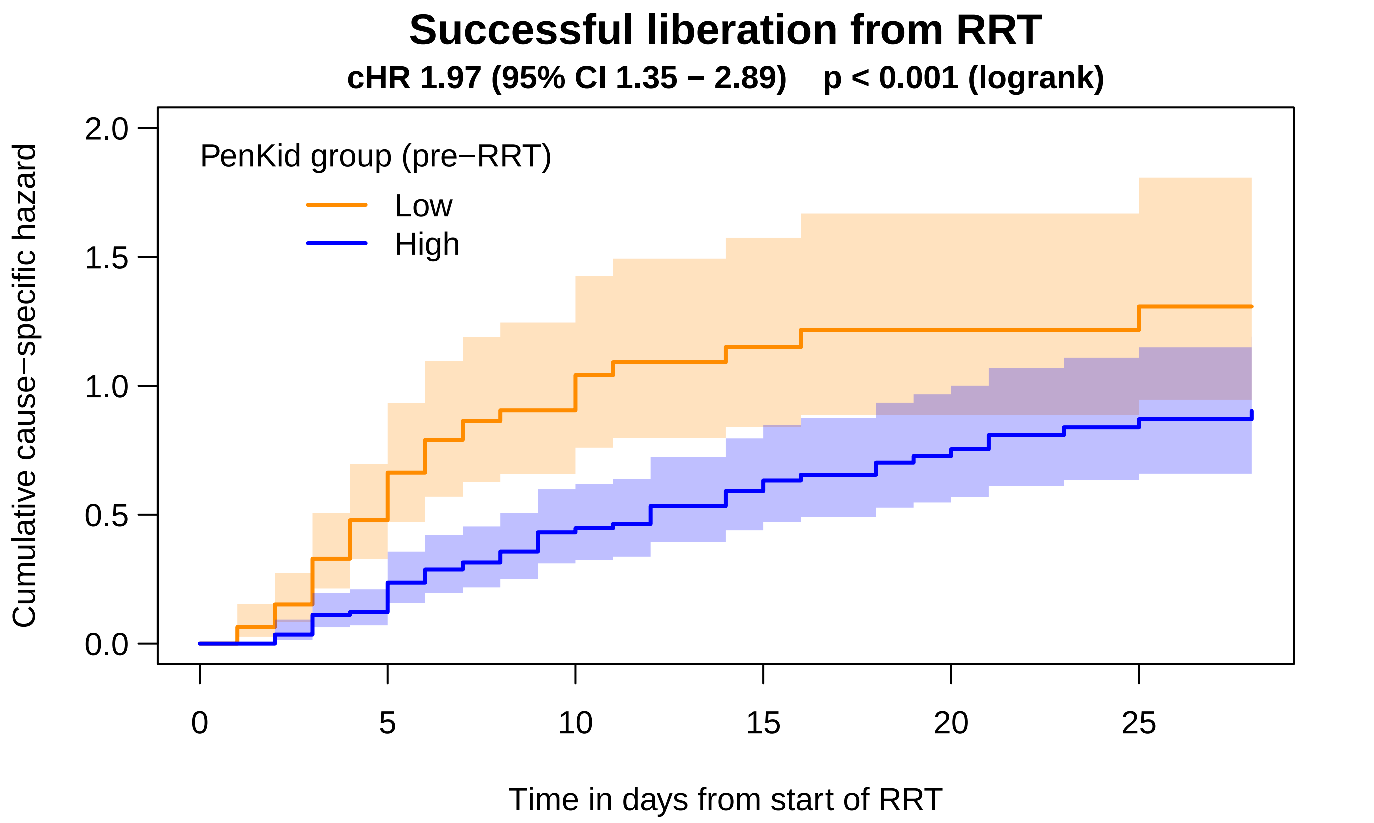
Successful liberation from RRT
cHR 1.97 (95% CI 1.35 − 2.89)   p < 0.001 (logrank)
PenKid group (pre−RRT)
Low
High
Cumulative cause−specific hazard
2.0
1.5
1.0
0.5
0.0
0
5
10
15
20
25
Time in days from start of RRT

(b)

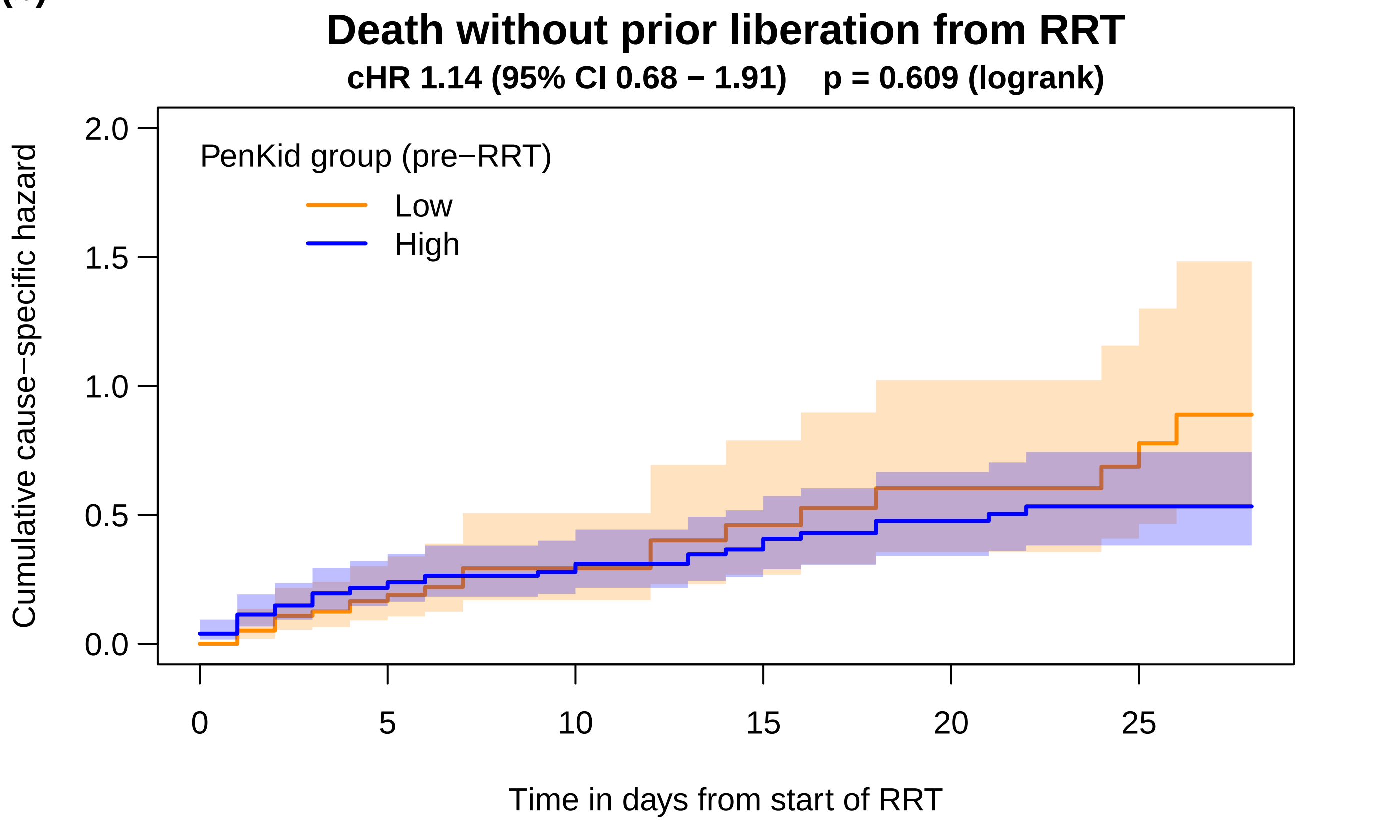
Death without prior liberation from RRT
cHR 1.14 (95% CI 0.68 − 1.91)   p = 0.609 (logrank)
PenKid group (pre−RRT)
Low
High
Cumulative cause−specific hazard
2.0
1.5
1.0
0.5
0.0
0
5
10
15
20
25
Time in days from start of RRT
