# Supplementary material for: Proenkephalin A 119–159 predicts early and successful liberation from renal replacement therapy in critically ill patients with acute kidney injury: a post hoc analysis of the ELAIN trial
Source: Crit Care. 2022 Oct 31;26:333. doi: 10.1186/s13054-022-04217-4 (PMC9624047; doi:10.1186/s13054-022-04217-4)
Supplement: Supplementary file 3 — Additional file 3. Figure S3: Estimated cumulative cause-specific hazard of successful liberation from RRT (a) and death without prior liberation from RRT (b) with log-transformed pointwise 95% confidence intervals, starting at the landmark on day 3 and including all patients still receiving RRT on the third day. [file 13054_2022_4217_MOESM3_ESM.pdf]

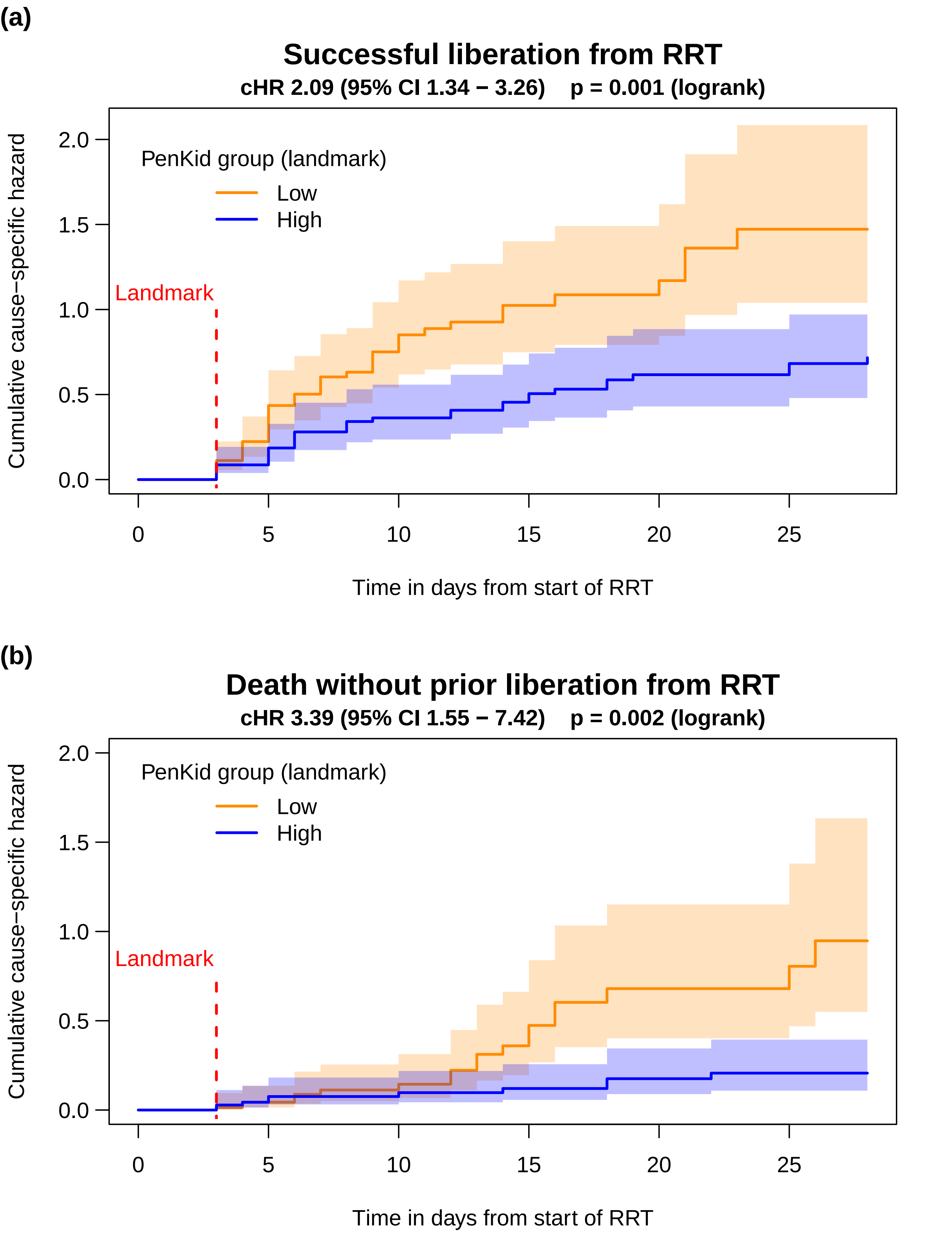
(a)
Successful liberation from RRT
cHR 2.09 (95% CI 1.34 − 3.26) p = 0.001 (logrank)
PenKid group (landmark)
Low
High
Landmark
Cumulative cause−specific hazard
2.0
1.5
1.0
0.5
0.0
0
5
10
15
20
25
Time in days from start of RRT
(b)
Death without prior liberation from RRT
cHR 3.39 (95% CI 1.55 − 7.42) p = 0.002 (logrank)
PenKid group (landmark)
Low
High
Landmark
Cumulative cause−specific hazard
2.0
1.5
1.0
0.5
0.0
0
5
10
15
20
25
Time in days from start of RRT
