# Supplementary material for: Proenkephalin A 119–159 predicts early and successful liberation from renal replacement therapy in critically ill patients with acute kidney injury: a post hoc analysis of the ELAIN trial
Source: Crit Care. 2022 Oct 31;26:333. doi: 10.1186/s13054-022-04217-4 (PMC9624047; doi:10.1186/s13054-022-04217-4)
Supplement: Supplementary file 9 — Additional file 9. Table S1: Patient characteristics of landmark patients, i.e., patients still receiving RRT at day 3. All variables were collected at the time of randomization except for estimated GFR, which was measured at the time of landmark penKid measurement. aFisher’s exact test comparing the low and high landmark penKid group. bMann–Whitney U test comparing the low and high landmark penKid group. Abbreviations: APACHE II, Acute Physiology and Chronic Health Evaluation II, GFR, Glomerular Filtration Rate; NGAL, Neutrophil gelatinase-associated lipocalin; SD, Standard Deviation; SOFA, Sequential Organ Failure Assessment [file 13054_2022_4217_MOESM9_ESM.pdf]

Variable	Total (n=169)	Low penKid (≤89 pmol/l) (n=75)	High penKid (>89 pmol/l) (n=72)	p-value	Missing penKid (n=22)
**Sex**					
Male	108 (63.9%)	50 (66.7%)	48 (66.7%)	1.000[a]	10 (45.5%)
Female	61 (36.1%)	25 (33.3%)	24 (33.3%)		12 (54.5%)
**Age (years)**					
Median (Q1, Q3)	69.0 (58.0, 75.0)	69.0 (59.0, 73.5)	70.0 (55.8, 77.0)	0.489[b]	69.5 (63.5, 74.0)
Mean (SD)	65.7 (13.5)	65.8 (11.6)	65.5 (15.3)		66.0 (14.1)
**Creatinine (mg/dl)**					
Median (Q1, Q3)	1.10 (0.800, 1.40)	1.00 (0.800, 1.28)	1.20 (0.900, 1.55)	0.010[b]	0.950 (0.800, 1.23)
Mean (SD)	1.14 (0.414)	1.06 (0.393)	1.25 (0.430)		1.02 (0.339)
Missing	4 (2.4%)	1 (1.3%)	1 (1.4%)		2 (9.1%)
**Estimated GFR (ml/min/1.73m^2)**					
Median (Q1, Q3)	53.0 (41.0, 80.5)	66.0 (55.0, 113)	42.5 (35.3, 50.0)	<0.001[b]	NA
Mean (SD)	73.1 (64.4)	95.7 (80.7)	49.7 (25.7)		NA
Missing	26 (15.4%)	2 (2.7%)	2 (2.8%)		22 (100%)
**SOFA score**					
Median (Q1, Q3)	14.0 (13.0, 16.0)	14.0 (13.0, 15.5)	14.0 (12.0, 16.0)	0.795[b]	14.5 (13.0, 16.0)
Mean (SD)	14.0 (2.80)	14.1 (2.54)	14.1 (2.93)		13.8 (3.28)
**APACHE II**					
Median (Q1, Q3)	25.0 (20.0, 30.0)	24.0 (20.0, 28.5)	25.0 (19.0, 31.3)	0.553[b]	26.5 (21.0, 34.5)
Mean (SD)	25.6 (7.39)	25.0 (6.64)	25.7 (7.98)		27.1 (7.93)
**Hypertension**					
Yes	140 (82.8%)	67 (89.3%)	58 (80.6%)	0.168[a]	15 (68.2%)
No	29 (17.2%)	8 (10.7%)	14 (19.4%)		7 (31.8%)
**Diabetes**					
Yes	31 (18.3%)	12 (16.0%)	13 (18.1%)	0.828[a]	6 (27.3%)
No	138 (81.7%)	63 (84.0%)	59 (81.9%)		16 (72.7%)
**Chronic obstructive pulmonary disease (COPD)**					
Yes	25 (14.8%)	16 (21.3%)	7 (9.7%)	0.069[a]	2 (9.1%)
No	144 (85.2%)	59 (78.7%)	65 (90.3%)		20 (90.9%)
**Vasopressors**					
Yes	152 (89.9%)	69 (92.0%)	64 (88.9%)	0.583[a]	19 (86.4%)
No	17 (10.1%)	6 (8.0%)	8 (11.1%)		3 (13.6%)
**Kidney biomarker NGAL (ng/ml)**					
Median (Q1, Q3)	557 (381, 935)	576 (368, 1040)	559 (403, 809)	0.653[b]	545 (379, 955)
Mean (SD)	671 (361)	695 (384)	648 (326)		663 (396)
